# Editorial: Impact of oxidation on nutrition: source, absorption and health effects

**DOI:** 10.3389/fnut.2025.1760719

**Published:** 2026-01-29

**Authors:** Małgorzata B. Różanowska, Mateusz Maciejczyk

**Affiliations:** 1 Cardiff Institute of Tissue Engineering and Repair, Cardiff University, Cardiff, Wales, United Kingdom; 2 School of Optometry and Vision Sciences, Cardiff University, Cardiff, Wales, United Kingdom; 3 Department of Hygiene, Epidemiology and Ergonomics, Medical University of Białystok, Białystok, Poland

**Keywords:** antioxidants, carotenoids, fatty acids, iron, oxidative stress, prooxidants, vitamin C, vitamin E

Nutritional science increasingly recognizes the importance of understanding how oxidative stress impacts health. This Research Topic attracted 10 contributions, nine of which are based on the analysis of health-related data gathered in the National Health and Nutrition Examination Survey (NHANES) from a representative sample of the U.S. population through in-person interviews and medical examinations (1).

Eight of these NHANES-based studies examined potential associations between the oxidative balance score (OBS) and various diseases and mortality in adults aged 20 years and older. Dietary OBS was calculated based on intakes of 16 dietary components (dietary fiber, carotenoids, riboflavin, niacin, vitamin B6, total folate, vitamin B12, vitamin C, vitamin E, calcium, magnesium, zinc, copper, selenium, total fat, and iron) evaluated from 24-h dietary recall interviews. The lifestyle OBS was based on four factors: a questionnaire assessing physical activity and alcohol intake, a measured body mass index (BMI), and a blood cotinine concentration as a marker of smoking. Dietary and lifestyle factors were divided, separately for males and females, into tertiles, where the antioxidant factors were scored 0 for the lowest tertile, 2 for the highest tertile and 1 for the middle tertile, whereas the prooxidant factors (dietary fat and iron, alcohol, high BMI and cotinine) were scored in reverse, with the highest tertile getting a score of 0 while the lowest tertile getting a score of 2. The associations between the conditions of interest and summed up OBS scores were analyzed by multivariate logistic regression models, without and with relevant covariates, from which the odds ratios and 95% confidence intervals were derived.

Sun et al. investigated associations between OBS and osteosarcopenia based on three cycles of NHANES where both the muscle mass and bone mass density (BMD) measurements were available (2005–2006, 2013–2014, and 2017–2018). BMD and appendicular skeletal muscle mass were assessed using dual-energy X-ray absorptiometry. The 3,336 eligible participants were classified into four groups: robust (no low BMD and no sarcopenia), low BMD alone, sarcopenia alone, and osteosarcopenia. Negative associations were found between total OBS and sarcopenia and osteosarcopenia in unadjusted and covariate-adjusted models. While sarcopenia was negatively associated with both dietary and lifestyle OBSs, there was no statistically significant association between osteosarcopenia and dietary OBS when adjusted for covariates.

Wang and Shi analyzed data of 3,625 participants diagnosed with the metabolic syndrome (MetS) in NHANES datasets spanning 1999–2006 and 2011–2018, for whom the sarcopenia assessment, OBS and relevant covariates data were available. The associations of sarcopenia with dietary OBS, lifestyle OBS and total OBS were investigated using three models without and with adjustments for different covariates. In all three models, all three OBSs were inversely associated with sarcopenia in the participants who were 40 years of age and older. It was shown that being in the highest quartile of OBS decreased the risk of sarcopenia by 51% in comparison with the lowest quartile.

Liang et al. examined associations between OBS and kidney disease in 3669 participants with type 2 diabetes using NHANES data from 2007 to 2018, whereas Huang et al. (2025) investigated associations between dietary OBS and kidney disease in 3,218 diabetic participants from 2001 to 2010 cohorts. Both studies showed a beneficial effect of higher dietary OBS on kidney function. In addition to the dietary OBS, Liang et al. also demonstrated negative associations between diabetic kidney disease and lifestyle and total OBSs.

Zhang and Yang used NHANES data from the period 2017–2020 to investigate associations between gallstones and total, dietary and lifestyle OBS. Gallstones were determined by a positive response to the question of whether the doctor told the participant they had gallstones. After exclusion of pregnant participants and participants without a gallstone questionnaire or incomplete OBS data, the analysis was performed based on 7,618 participants. Higher OBS was associated with lower gallstones risk, particularly in participants who were under 60 years of age, Hispanic, with hypertension or malignancy. It has been shown that the dietary and lifestyle OBS independently contribute to the protection against gallstones when adjusted for age, gender, race and educational level.

Zhang et al. investigated the associations of OBS with mortality and insulin resistance in the cohort of 11,849 adults from NHANES 2007–2018. The probability-matching algorithm was used to extract mortality data from the NHANES mortality file, updated through the end of 2019, and from the National Death Index. There was a negative association of all-cause mortality with the OBS when adjusted for all covariates, with participants in the highest tertile of OBS having a reduced risk by about 35% in comparison with the lowest tertile. This negative association was even more pronounced for cardiovascular mortality. No statistically significant associations were found between OBS and cancer mortality. Several markers of insulin resistance were negatively associated with OBS and positively associated with all-cause and cardiovascular mortality but only for participants below the age of 65 years.

Wu, Dong et al. examined associations between OBS and thyroid dysfunction based on NHANES data of 6,268 participants from the 2007–2012 cohorts and found a negative association between OBS and subclinical hyperthyroidism (SCHyper), even after adjusting for covariates, but there were no statistically significant associations of OBS with other thyroid conditions, such as hyperthyroidism, Hashimoto's thyroiditis, autoimmune thyroiditis subclinical hypothyroidism and hypothyroidism. Wu, Niu et al. investigated the same NHANES 2007–2012 cohorts for the association of thyroid dysfunction with the intake of selenium, zinc, and total carotenoids, and vitamins A, C, and E. While before the adjustments for covariates, each of these dietary factors showed a negative association with SCHyper, none of them remained statistically significant after the adjustments. However, when the effect of the six nutritional components were transformed into the composite dietary antioxidant index (CDAI), the negative association between CDAI and SCHyper was significant after adjustments for all covariates.

Hu, Chen et al. examined the potential association between migraine or severe headache and serum levels of six carotenoids: α-carotene, β-carotene, β-cryptoxanthin, lutein/zeaxanthin, and lycopene, collected in NHANES 2001–2004. The migraine/severe headache was determined based on an affirmative answer to the question in the pain questionnaire: “Have you experienced severe headaches or migraines in the past 3 months?” The prevalence of severe headache/migraine was 22% and was negatively associated with serum total carotenoid levels, as well as with all individual carotenoids except lycopene.

The only contribution that was not based on NHANES data was by Hu, Yue et al., who provided a brief overview of literature investigating the effect of various food-derived antioxidants on *in vitro* and *in vivo* models of metabolic dysfunction-associated fatty liver disease (MAFLD), as well as on the effects of some of them in clinical trials on MAFLD patients, and prospective and cross-sectional studies of the association of OBS with MAFLD. While a growing body of evidence suggests a beneficial effect of various antioxidants in ameliorating MAFLD, in most cases, there remains a lack of randomized controlled clinical trials demonstrating their efficacy in patients.

Altogether, the publications mentioned above contribute to the growing body of evidence on the crucial roles of dietary antioxidants, and sometimes also prooxidants, in modulating susceptibility to various diseases.
